# Erratum for Mirhendi et al., the first case of onychomycosis in a koala *(Phascolarctos cinereus) *due to atypical isolates of *Microsporum gypseum*, a diagnostic challenge

**DOI:** 10.18502/cmm.4.1.34

**Published:** 2018-03

**Authors:** H Mirhendi, Y Nishiyama, A Rezaei-Matehkolaei, K Satoh, K Makimura

**Affiliations:** 1 Department of Medical Parasitology & Mycology, School of Medicine, Isfahan University of Medical Sciences, Isfahan, Iran; 2 Department of Medical Parasitology & Mycology, School of Public Health, Tehran University of Medical Sciences, Tehran, Iran; 3 Teikyo University Institute of Medical Mycology, Tokyo, Japan; 4 Department of Medical Mycology, School of Medicine, Health Research Institute, Infectious and Tropical Diseases Research Center, Ahvaz Jundishapur University of Medical Sciences, Ahvaz, Iran; 5 General Medical Education and Research Center, Teikyo University, Tokyo, Japan

**ERRATUM**

Volume 2, no. 2, 45-50, 2016, DOI: 10.18869/acadpub.cmm.2.2.2 [1]. The original version of this article contained incorrect dose of terbinafine. This erratum adds the correct dose as shown below. 

Terbinafine dosage should change from 250 mg/kg to 250 mg/day.
